# Development of novel genic microsatellite markers from transcriptome sequencing in sugar maple (*Acer saccharum* Marsh.)

**DOI:** 10.1186/s13104-017-2653-2

**Published:** 2017-08-08

**Authors:** Monica Harmon, Thomas Lane, Margaret Staton, Mark V. Coggeshall, Teodora Best, Chien-Chih Chen, Haiying Liang, Nicole Zembower, Daniela I. Drautz-Moses, Yap Zhei Hwee, Stephan C. Schuster, Scott E. Schlarbaum, John E. Carlson, Oliver Gailing

**Affiliations:** 1 School of Forest Resources and Environmental Science, Michigan Technological University, 1400 Townsend Drive, Houghton, MI 49931 USA; 2 Department of Entomology and Plant Pathology, The University of Tennessee, Knoxville, TN 37996 USA; 3 Department of Forestry, University of Missouri, Columbia, MO 65274 USA; 4 Department of Ecosystem Science and Management and Department of Plant Science, The Pennsylvania State University, University Park, PA 16802 USA; 5 Department of Genetics and Biochemistry, Clemson University, Clemson, SC 29630 USA; 6 College of Agriculture, Fisheries and Forestry, Fiji National University, Nausori, Fiji; 7 Singapore Centre for Environmental Life Sciences Engineering, Nanyang Technological University, Singapore, 637551 Singapore; 8 Department of Forestry, Wildlife & Fisheries, The University of Tennessee, Knoxville, TN 37996-4563 USA; 9 Forest Genetics and Forest Tree Breeding, Faculty of Forest Sciences, University of Goettingen, Buesgenweg 2, 37077 Goettingen, Germany

**Keywords:** *Acer saccharum*, EST-SSRs, Transferability, Next-generation sequencing

## Abstract

**Background:**

Sugar maple (*Acer saccharum* Marsh.) is a hardwood tree species native to northeastern North America and economically valued for its wood and sap. Yet, few molecular genetic resources have been developed for this species to date. Microsatellite markers have been a useful tool in population genetics, e.g., to monitor genetic variation and to analyze gene flow patterns. The objective of this study is to develop a reference transcriptome and microsatellite markers in sugar maple.

**Findings:**

A set of 117,861 putative unique transcripts were assembled using 29.2 Gb of RNA sequencing data derived from different tissues and stress treatments. From this set of sequences a total of 1068 microsatellite motifs were identified. Out of 58 genic microsatellite markers tested on a population of 47 sugar maple trees in upper Michigan, 22 amplified well, of which 16 were polymorphic and 6 were monomorphic. Values for expected heterozygosity varied from 0.224 to 0.726 for individual loci. Of the 16 polymorphic markers, 15 exhibited transferability to other *Acer* L. species.

**Conclusions:**

Genic microsatellite markers can be applied to analyze genetic variation in potentially adaptive genes relative to genomic reference markers as a basis for the management of sugar maple genetic resources in the face of climate change.

**Electronic supplementary material:**

The online version of this article (doi:10.1186/s13104-017-2653-2) contains supplementary material, which is available to authorized users.

## Background

Sugar maple (*Acer saccharum* Marsh.) is a hardwood species native to the Northeastern United States and Southern Canada. It is valued for its wood and syrup/sugar, supports a large industry [1] and is an important ecosystem component [2]. While microsatellite markers have many applications in population genetic analyses, few resources for sugar maple have been developed to date [3, 4].

Microsatellite markers are short sequence repeats (SSRs) which are often highly variable and prone to mutations [5]. Genic microsatellite markers can be derived from EST-libraries and can be located in the coding regions or in the 5′- and 3′ untranslated regions of mRNAs [6]. Due to their location in expressed genes, they often show a lower genetic variation than genomic (non-genic) microsatellites and a higher transferability between related species [6]. Especially, stress-responsive genes might be under selection and show clinal variation along environmental gradients. Heat and drought stress and population fragmentation are especially expected at the southern distribution edge of the species [7], as the result of a warming climate. The new genic microsatellites will be useful to analyze patterns of genetic variation and provide the basis for the management and conservation of the species’ genetic resources in a changing climate.

## Methods

### Plant materials for SSR analysis

Leaf samples were collected from 47 georeferenced adult trees of one natural population close to Houghton, MI (47°07′07.46″N, 88°35′16.41″W) in a region which is dominated by sugar maple forests [4]. Total genomic DNA was isolated for the 47 samples of *Acer saccharum* and for three samples of each *Acer rubrum* L. (section *Rubra*), *Acer saccharinum* L. (section *Rubra*), *Acer platanoides* L. (section *Platanoidea*) and *Acer ginnala* Maxim. (section *Ginnala*) from ~1 cm^2^ of silica gel dried leaf samples using the DNeasy Plant Mini Kit (Qiagen, Hilden, Germany).

### Library preparation, transcriptome sequencing and assembly

A total of 51 transcriptome libraries were derived from tissues (leaves, petioles, roots) of ozone, cold, heat, drought, wound stressed and unstressed half-sib seedlings. Tissue types and treatments are described in Additional file 1. Details on ozone and wound stress treatments are given in [8]. Tissues were flash frozen and stored at −80 °C until RNA was extracted. Approximately 1 g of frozen tissue was used for isolation of total RNA with a modified CTAB method that uses lithium chloride precipitation [9]. After RNA quality assessment with an Agilent Bioanalyzer (Agilent Technologies), RNA was subjected to TruSeq Stranded mRNA library preparation (San Diego, CA), following the manufacturer’s protocol. All libraries were labeled with dual barcodes. Paired-end sequencing of 46 sugar maple libraries was performed on an Illumina HiSeq 2500 instrument (San Diego, CA) at a read length of 101 bp. A further set of 5 libraries were sequenced on an Illumina MiSeq (San Diego, CA) (Additional file 1). Raw reads can be found in the NCBI Short Read Archive (SRA) with the bioproject accession PRJNA273272. Read statistics for each library are provided in Additional file 1.

Trimmomatic version 0.35 was used to trim raw sequencing reads of adapter sequences and low quality bases [10]. Kmer-based error correction of RNASeq reads was performed in Rcorrector (kmer length of 31) [11]. Trimmed and corrected reads were assembled with the Trinity pipeline version r20131110 [12]. Isoforms from the Trinity pipeline were collapsed and further assembled with cd-hit version 4.6.1 [13]. Open reading frame (ORF) prediction was executed with TransDecoder version 2.0.1 [14]. TransDecoder predictions were further improved by performing searches using BLAST version 2.2.29 versus the Uniprot SwissProt protein databases (cut-off of e-value <1e−4) and by HMMER version 3.1b2 against the pfam database [15–18]. Two methods were employed to determine assembly completeness of the sugar maple transcriptome. First, transcripts were assessed by BUSCO (Benchmarking Universal Single-Copy Orthologs) version 1.1b1 to determine the presence of universal single-copy orthologs using the early release plant database [19]. Second, TransRate v1.0.3 was used to calculate quality scores for contigs based on the read alignment evidence [20].

### Functional annotation and SSR discovery

BLAST version 2.2.29 queries were performed against the plant taxonomic division of the TrEMBL protein database and Swiss-Prot protein database [15, 16]. Simple sequence repeats (SSRs) were found by searching putative unique transcripts (PUTs) using a perl script (https://github.com/mestato/lab_code/tree/master/hwg_gssr_scripts). SSRs needed to meet specific requirements for inclusion in the final output, requiring: 2 bp repeats to have 8–200 copies, 3 bp repeats to have 7–133 copies, and 4 bp repeats to have 6–100 copies. Compound SSRs, defined as SSRs occurring with less than 15 bases of separation, were removed. Dustmasker was used to mask low complexity regions to aid primer design in Primer3 v2.3.6 [15, 21]. The following Primer3 parameters were used: primer_product_size_range  =  100–450, primer_min_tm  =  55.0, primer_max_tm  =  65.0, primer_min_gc  =  40, primer_max_gc  =  60, primer_max_poly_x  =  3, primer_gc_clamp  =  2.

### Marker analyses

The DNA was diluted to ~1 ng/µl with double deionized water (Ultra Pure Water, Molecular Grade, Phenix Research Products). A 15 µl PCR reaction was prepared consisting of 6 µl double deionized water (Ultra Pure Water, Molecular Grade from Phenix Research Products), 5 µl HotFIREPol (Solis Biodyne, containing 2 mM dNTPs, 1U Taq polymerase, 10 mM MgCl_2_), 0.2 µl of 5 µM forward primer with the M13 tail (5′-CACGACGTTGTAAACGAC-3′), 1.5 µl of 5 µM 5′ dye labelled (6-FAM) M13 primer, 0.5 µl of 5 µM pig-tailed (5′-GTTTCTT-3′) reverse primer [22, 23] (Sigma Aldrich Inc., St. Louis, MO) and 2 µl DNA (~2 ng). The PCR touchdown reaction was performed in an Applied Biosystems 2720 Thermal Cycler (Foster City, CA) consisting of an initial denaturation at 95 °C for 15 min, 10 touch-down cycles of 1 min at 94 °C, 1 min at 60 °C (decreasing 1 °C each cycle), and 1 min at 72 °C, followed by 25 cycles of 94 °C for 1 min, 50 °C for 1 min and 72 °C for 1 min. The final extension was at 72 °C for 20 min. PCR products were checked on 1.5% agarose gels and stained with 2 µl GelRed (10,000X in water; Biotium, Hayward, CA) and exact fragment sizes were determined after electrophoretic separation on an ABI Prism^®^ Genetic Analyzer 3730 with Gene-ScanTM LIZ-500 as internal size standard. Alleles were assigned to bins after careful visual inspection using GeneMarker^®^ V2.6.7 (SoftGenetics).

Primer pairs (Sigma Aldrich Inc., St. Louis, MO) were tested for amplification initially in four randomly selected samples. Markers that amplified polymorphic products in the expected size range in these four samples were amplified in all 47 samples. Genetic variation assessment was conducted using GenAlEx 6.502 [24]. Specifically, observed and expected heterozygosity (H_o_, H_e_, respectively) [25] and number of alleles (N_a_) were calculated. Further, the inbreeding coefficient (*F*) was estimated for each locus in the population using GENEPOP version 4.2 [26, 27]. Significant deviations from Hardy–Weinberg proportions were tested using Fischer’s exact test in GENEPOP. Additionally pairwise linkage disequilibrium was tested for all marker pairs in GENEPOP. Bonferroni corrections were applied to adjust for multiple testing [28].

## Results and discussion

### Transcriptome assembly and validation

Illumina sequencing of 51 transcriptome sugar maple libraries of varying tissue types and treatments produced 282 million reads (29.9 Gb), available at NCBI SRA bioproject accession PRJNA273272 (Additional file 1). De novo assembly of reads yielded 117,861 putative unique transcripts (PUTs) with an average length of 945 bases and an N50 length of 1667 bases. TransDecoder version 2.0.1 was used to identify 67,537 possible open read frames (ORFs) derived from 51,390 PUTs (43.6%). Multiple ORFs per PUT can occur for example due to chimeras in the assembly or sequencing errors (insertions/deletions) that shift the reading frame incorrectly. The peptide sequences calculated from the ORFs average 284 amino acids in length. Assembly accuracy was assessed as quality metrics based on examination of read mapping using Transrate [20] and identified an overall mapping rate of 72.8%, and over 85% of those mapped reads supported the assembly (i.e. both pairs aligned to the same transcript in the correct orientation without overlapping either transcript end). Out of a total of 956 BUSCO ortho-groups searched, 914 were found in the BUSCO database. These were further classified as 434 complete single-copy genes, 446 complete but duplicated genes, and 34 fragmented copies of genes. This represents the first transcriptome reference for sugar maple and will be a valuable genomic resource for future studies, for example, to generate markers, to use as a reference for gene expression sequencing or to identify candidate stress-response genes. Raw reads are stored at NCBI Short Read Archive (SRA) with the bioproject accession PRJNA273272.

### Functional annotation and SSR discovery

Sugar maple PUTs were used as queries for BLAST searches against the proteins in the Swiss-Prot and plant TrEMBL [16] databases yielding 64,458 (54.7%) PUTs with matches to TrEMBL plant proteins and 49,760 (42.2%) PUTs with matches to Swiss-Prot. Simple Sequence Repeat (SSR) extraction found 6279 SSRs in 5965 PUTs (5.1%). SSRs were identified for 2, 3, and 4 bp motifs. The 4 bp motifs were the rarest, making up 0.97% of total SSRs found. The shorter 2 bp and 3 bp motifs were much more common, making up 78.7 and 20.4% of total SSRs found, respectively. Primers were designed for 1068 SSRs using the flanking regions of the repeats (Additional file 2). Of these 812 were 2 bp repeats ranging from 8 to 15 repeats, 234 were 3 bp repeats ranging from 7 to 14 repeats, and 22 were 4 bp repeats with 6 repeats. Additional file 2 contains a report of the SSR analysis along with primers designed for SSRs meeting the appropriate requirements and functional annotations for SSR markers.

### Microsatellite marker characterization

We selected 58 SSRs with a wide range of expected amplicon sizes (100–487 bp) to develop sets of markers with non-overlapping size ranges that could be used for multiplex PCRs in subsequent studies. Out of the 58 primers tested, 16 amplified polymorphic loci and 6 amplified monomorphic loci (Table 1; Additional file 2). The expected size for the polymorphic markers varied from 113 and 425 bp. For polymorphic loci H_e_ ranged from 0.224 to 0.726, H_o_ from 0.143 to 0.722 and N_a_ from 2 to 12. A total of 11 out of the 16 markers showed no significant deviation from Hardy–Weinberg proportions after Bonferroni correction, while markers As_di12 and As_di38 (p < 0.05) and markers As_di34, As_di48 and As_di9 (p < 0.01) showed significant and positive F values. No significant linkage disequilibrium was found between marker pairs after Bonferroni correction (p < 0.05).

**Table 1:** Primer sequences and descriptions of 22 microsatellite markers developed in *Acer saccharum*

Locus	Primer sequences (5′–3′)	Repeat EdEdmotif	Size range (bp)	**Expected fragment size	N_a_	H_o_	H_e_	F	*p*
As_di1	F: TCCCAGGCATGAACAAGGTT R: TGCAGTAAGTTGACAGCTCT	(AC)_9_	251–273	230	9	0.581	0.658	0.117	0.0034
As_di7	F: GGGTCTGTCTCTGTTTCTGCA R: ACAGGGTTCACTGAGCTGTG	(AC)_9_	151–161	134	6	0.651	0.602	0.081	0.2578
As_di9^a^	F: TGCTGGAAAGTGGAACCTGT R: AGTCTGATCTGTCATGGGCTC	(TA)_8_	121–147	100	12	0.375	0.835	0.551	<0.0001**
^m^As_di11	F: AGAGAACCACCAAGGATGCA R: CAGGGAGCCATTTCACTCTGA	(GA)_8_	187	167	1	0	0	–	–
As_di12	F: AAGACATCTTGAGGGCGGTG R: TGTAACTGCATAACGGGCCA	(TC)_9_	447–458	425	4	0.194	0.271	0.283	0.0014*
^m^As_di13	F: TCAAGAAATACTGGCTCAGGTCA R: ACATGCATGTTGAGCGATTGT	(TC)_8_	264	238	1	0	0	–	–
As_di15	F: GGGCAGAGAGGGAATTCGAG R: TGGGGAGACAGAACTTGTGC	(TC)_8_	259–265	235	4	0.375	0.429	0.126	0.0236
As_di16	F: AATTGCCTGTGGTGGGAACT R: ACTCCACTCTCTTTCTTGCTCA	(GT)_8_	211–218	190	4	0.675	0.680	0.007	0.8696
^m^As_di19	F: GACCTGACCACCTCCTCCTA R: AGGGCAACATACACGGATCG	(CT)_9_	228	205	1	0	0	–	–
As_di21	F: TGTCAGCAGCCCTACAGTTG R: ACAGGTCACGATCTCTCCCT	(GT)_8_	135–142	113	4	0.450	0.609	0.261	0.0455
^m^As_di27	F: TCATGACCATGACCCAACACT R: TCCTGTGGATTCTTTTGTATCTGGT	(TC)_8_	386	363	1	0	0	–	–
^m^As_di30	F: GATCCCCTTCGTTGCTGACA R: GGACTGCCCGATTGATACTCA	(TG)_9_	457	432	1	0	0	–	–
^m^As_di31	F: CTCCACCACCATCCAACCAA R: TCCTCAGCTTCTTGGGCTTG	(AC)_8_	187–189	167	1	0	0	–	–
As_di34^a^	F: AACGGATGGCAAGCTAGCTT R: CAGGCCTGCCCAGAAACTAA	(AC)_8_	225–240	218	4	0.217	0.726	0.701	<0.0001**
As_di35	F: TGTTAGTCTCCTCCACACGT R: CAGCAGCAGCAGCAAAACT	(TC)_8_	151–153	129	2	0.143	0.224	0.362	0.0751
As_di36	F: ATGTGAGTCCGTGAGTCCGT R: ATAAGCAGCAGCAGCAGACA	(TG)_8_	237–245	212	5	0.475	0.606	0.216	0.0322
As_di37	F: TGGTGGGTAGCAGCAAAAGA R:TGCACATGGGATGATGATCAGT	(TG)_11_	167–184	151	8	0.722	0.704	−0.025	0.9324
As_di38	F: ACAGAGAGAGAGAGAGCTTGT R: TACCGTCATGGCCGATGATG	(AC)_8_	175–179	154	3	0.184	0.362	0.491	0.0012*
As_di41	F: AAGCTGAGAAACCCAAAGCA R: CACCACCCAACCCTTTTCCT	(TA)_8_	259–271	236	6	0.526	0.625	0.157	0.2675
As_di48	F: AGGTTCGGGTTTTGAATCTTCA R: CAAGGACTTTGGCTCTGCTG	(TA)_8_	173–179	155	4	0.200	0.678	0.705	<0.0001**
As_di49	F: TGCAACTGTTGAGTGGTGGA R: ACAAGTCGAACAACCCGTTG	(TG)_10_	159–183	133	10	0.625	0.636	0.017	0.0528
As_tetra1	F: TTGACGGAGAGCTTGGTTCC R: AAAACCCAATTCGCCACGTG	(TGCT)_6_	257–273	241	5	0.341	0.337	−0.012	0.4674
^+^Mean					6	0.424	0.543	0.222	
Mean					4	0.308	0.395	0.222	

*N*_*a*_ number of alleles per locus, *H*_*o*_ observed heterozygosity, *H*_*e*_ expected heterozygosity, *F* inbreeding coefficient

* Significantly different from Hardy–Weinberg proportions (α = 0.05) after Bonferroni corrections

** Significantly different from Hardy–Weinberg proportions (α = 0.01) after Bonferroni corrections ^m^: these markers amplified monomorphic loci and were not tested on the whole population. **: expected fragment size based on EST contigs. Actual fragments were longer as tailed primers were used for amplification. ^+^Mean variation was calculated for polymorphic markers only to allow for comparisons with other related studies on sugar maple [4]

^a^ Markers As_di34 and As_di9 did not amplify in nearly half of the samples and should not be used for population level analyses

A total of 16 markers were tested for transferability in three DNA samples from each of the four *Acer* species which represent three taxonomic sections: *A. saccharinum* (section *Rubra*), *A. rubrum* (section *Rubra*), *A. platanoides* (section *Platanoidea*) and *A. ginnala* (section *Ginnala*) using the same PCR protocol as described above. The results showed that most primers amplified a multi-banding pattern across species. However, 15 markers amplified single loci in the expected size range in at least one other *Acer* species (Table 2). Ten markers were monomorphic in some or all species (Table 2). Two markers, As_tetra1 and As_di41, were polymorphic in more than one species.

**Table 2:** Transferability of *Acer saccharum* microsatellite loci to other *Acer* species

*A. saccharum*	*A. rubrum*	*A. saccharinum*	*A. platanoides*	*A. ginnala*
As_di1	–	–	As_di1	As_di1*
As_di7	As_di7	As_di7*	–	–
As_di9	–	–	As_di9	As_di9*
As_di12	–	As_di12	–	–
As_di15	–	–	As_di15	As_di15*
As_di16	–	–	–	As_di16
As_di21	–	As_di21*	As_di21	–
As_di35	As_di35	As_di35*	As_di35*	–
As_di36	–	–	As_di36*	–
As_di37	–	As_di37*	As_di37*	–
As_di38	–	As_di38	–	–
As_di41	As_di41	As_di41	As_di41	As_di41
As_di48	As_di48	As_di48*	As_di48*	As_di48*
As_di49	–	–	As_di49	As_di49*
As_tetra1	–	–	As_tetra1	As_tetra1

All of these markers amplified single polymorphic loci in *A. saccharum*

* Monomorphic in species as indicated by column. Only markers that are transferable to at least one of the four maple species are shown

– No amplification

The level of genetic variation for individual polymorphic markers varied considerably. Mean variation at polymorphic markers was much lower for genic (H_o_:0.424; H_e_:0.543) than for non-genic markers in the same samples (H_o_:0.708; H_e_:0.822 [4]). Similarly, lower genetic variation was observed at genic EST-SSRs than at genomic SSRs in several *Quercus rubra* L. and *Quercus ellipsoidalis* E.J. Hill populations [29]. The generally lower genetic variation at genic EST-SSRs suggests that these markers are not selectively neutral, but subject to selection [6]. Genetic variation observed at genic EST-SSRs in sugar maple was lower than estimates obtained in other wind-pollinated tree species such as North American oak species *Q. rubra* and *Q. ellipsoidalis* from the same geographic region (H_e_ = 0.700, range 0.690–0.740, [29]). As for non-genic markers which were characterized in the same population [4] most F values are not significantly different from zero. High F values at individual markers could be due to the presence of null alleles. Markers As_di34 and As_di9 with high F values and significant deviation from Hardy–Weinberg proportions (p < 0.001) showed no amplification in nearly half of the samples suggesting high null allele frequencies. All other markers amplified in most samples making them applicable for population genetic analyses. Only two out of the 47 samples showed variable amplification success potentially as result of low DNA quality. The other marker, As_di48, with a very high F value showed high amplification success comparable to the other markers. Overall high variation was observed in F values ranging from −0.081 for As_di7 to 0.705 for As_di48.

The overall high level of genetic variation, the absence of linkage disequilibrium and low to moderate deviations from HWE for most markers suggest the suitability of these markers for population genetic studies in sugar maple. In addition, markers showed high transferability to other *Acer* species providing at least two new polymorphic markers for the four other *Acer* species, and even six new polymorphic markers for one of the species, *A. platanoides.* High transferability of markers across *Acer* sections suggests their potential usefulness for population genetic analyses in other maple species. In contrast, only two out of 13 genomic SSRs amplified polymorphic loci in one other species, *Acer ginnala* [4]. This high transferability is typical of EST-SSRs and has been shown repeatedly in crop plants and is more prevalent than in non-EST-SSR markers [6]. Genetic variation at SSR markers was only assessed in a single natural population in Michigan. Allelic diversity will certainly vary among populations within the large distribution range of the species. However, in outcrossing, wind-pollinated tree species with wide and continuous distribution ranges such as sugar maple most of the genetic variation (i.e. H_e_) is generally distributed within populations [30] and similar levels of within population variation are expected within the main distribution range of the species.

## Conclusions

Especially in the trailing (southern) edges of the species’ distribution range lower genetic variation is expected as result of differential mortality due to increased abiotic and biotic stressors. With the availability of both neutral genomic SSRs and genic SSRs with potential role in adaptation, patterns of genetic variation and their relation to environmental and climatic variables can be studied. For example, the presented marker and transcriptome resources can be used to identify genes involved in the adaptation to these stressful conditions, for example by analyzing associations of alleles with environmental gradients [31].

## Additional files

**Additional file 1.** Description of the 51 sugar maple transcriptome libraries. Information on the stress treatment, tissue and number of HiSeq reads is given for each of the 51 transcriptome libraries.

**Additional file 2.** Summary report of all EST-SSRs. The summary statistics and primer sequences are given for all EST-SSRs. Gene annotations are provided for the newly developed EST-SSR markers.
